# Commoning, heterotopia, and transformation: An analytical framework for and from contested spaces

**DOI:** 10.1177/02637758251361706

**Published:** 2025-09-10

**Authors:** Amy R Poteete, Pavel Kunysz, Nik Luka

**Affiliations:** 1 Department of Political Science, 5618 Concordia University, Montreal, Canada; 2 26658 Faculty of Architecture, Unité de Recherche en Architecture (URA), University of Liège, Liège, Belgium; 3 Peter Guo-hua Fu School of Architecture + School of Urban Planning, 5620 McGill University, Montreal, Canada

**Keywords:** Commoning, heterotopia, social transformation, mutualization, denormalization, urban green space

## Abstract

Commoning occurs when people recognize that they share something and develop a sense of mutuality toward each other along with a shared responsibility for whatever they share. Because sharing and mutuality contrast with the individualism, competitiveness, and profit orientation of contemporary capitalist societies, commoning is widely heralded for its transformative potential. Nonetheless, commoning is not inherently transformative. We argue that whether commoning supports transformation depends on its relationship with heterotopic processes. Both commoning and heterotopia—ideal typical “other” spaces characterized by looseness and denormalization—present alternatives to hegemonic norms, especially those of state-centricity, hierarchical social organization, and the prioritization of market relationships and economic growth, but they are distinct processes that do not necessarily coincide. We propose an analytical framework to guide analysis of the relationship between commoning and heterotopia and illustrate it with examples from contested urban green spaces in Liège (Belgium), Montréal (Canada), and Brussels (Belgium).

## Introduction

Commoning occurs when a set of people comes to recognize that they share a place, space, thing, or project, and develop a sense of mutuality toward each other, along with shared responsibility for whatever it is that they share (Poteete et al., 2021; see also Bollier and Helfrich, 2015, 2019; Linebaugh, 2008). Because sharing and mutuality contrast sharply with the individualism, competitiveness, and emphasis on monetary values that characterize contemporary capitalist societies, commoning is widely heralded for its transformative potential (e.g. Bollier and Helfrich, 2019; Gibson-Graham et al., 2016; Sevilla-Buitrago, 2022; Stavrides, 2019; Varvarousis and Kallis, 2017). Whatever its promise, however, commoning is not inherently or inevitably transformative (Anderson and Huron, 2023; Caffentzis, 2010; Tummers and MacGregor, 2019; Velicu and García-López, 2018: 61). Indeed, commoning may instead reproduce and even reinforce the status quo, as often occurs when it is based on identities such as ethnicity, caste, or socioeconomic status (e.g. Nightingale, 2019) or where it fails to challenge systemic inequalities (Kashwan et al., 2021). Sometimes it favors new axes of inequality and exclusion that are at least partially compatible with the status quo, or supports processes of normalization, cooptation, and incorporation (e.g. Helten, 2015; Stavrides, 2019: 196–197). To better understand the variable relationships between commoning and transformation, we need to better understand how commoning interacts with other processes and conditions in specific contexts (cf. Velicu and García-López, 2018: 61).

Our central claim is that whether commoning fosters social, ecological, economic, and/or political transformation depends on its ongoing relationships with heterotopic processes. Foucault (2004 [1967]) introduced the concept of heterotopia as ideal typical “other spaces” (see also the essays in Dehaene and De Cauter, 2008). Designating a space as heterotopic signals diversity and perhaps deviance (Foucault, 2004 [1967]; cf. Doron, 2008), informality, “looseness” (little to no effective regulation or formal programming—see Franck and Stevens, 2007), “porosity” (fluidity or blurring of boundaries—see Stavrides, 2007), and/or dynamism—combined with what Lefebvre (1971, 1974) saw as possibilities for transformation. Both commoning and heterotopia present alternatives to hegemonic societal norms, especially those of state-centricity, hierarchical social organization, and the prioritization of market relationships and economic growth (inter alia, Bollier and Helfrich, 2019; Edwards and Bulkeley, 2018; Federici, 2019; Gibson-Graham et al., 2016; Helten, 2015; Kashwan et al., 2021; Stavrides, 2007). They are, however, distinct processes that neither necessarily coincide nor complement each other.

When state regulation and social norms recede, as they do under heterotopic conditions, possibilities for nonhierarchical, bottom-up social processes—including commoning—also expand (Arampatzi, 2022; Ellickson, 1991; Ostrom, 1990; Varvarousis and Kallis, 2017). Indeed, in urban settings, commoning often emerges in heterotopic spaces (Doron, 2008; Helten, 2015; Stavrides, 2007). Examples include squatting of vacant buildings, guerrilla gardening, outdoor raves, and innumerable social movements to prevent the normalization of spaces and places (e.g. Bresnihan and Byrne, 2015; Hess, 2008; Noterman, 2022; see other examples compiled in Bollier and Helfrich, 2015, 2019; Ratto and Boler, 2014). Commoning involves recognition of interdependency, mutualization, and the emergence of norms (Bollier and Helfrich, 2015, 2019; Linebaugh, 2008), whereas heterotopia is norm-defying and ambiguous (Doron, 2008; Edwards and Bulkeley, 2018; Stavrides, 2007). Commoning, thus, is not always heterotopic, and heterotopia does not inevitably give rise to or support the survival of commoning. We argue that commoning contributes to the vitality and durability of bottom-up mobilization to reclaim, share, and repurpose—transform!—all sorts of spaces, places, things, and resources when it interacts productively with heterotopic processes, but that it is more likely to reinforce the status quo in the absence of heterotopic processes or when in tension with such processes. Commoning and heterotopia and their articulation are therefore pivotal for the question of social transformation.

In this article, we develop an analytical framework for evaluating the presence of commoning and heterotopia and relationships among them—and thus possibilities for transformative commoning—in specific times and places. Our framework focuses on two processes associated respectively with commoning and heterotopia: mutualization and denormalization. As we illustrate with examples of contested urban green spaces in Liège (Belgium), Montréal (Canada), and Brussels (Belgium), this framework can be used to evaluate the relationship between commoning and heterotopia in specific times and places, as well as to track changes in this relationship over time. By distinguishing between heterotopic and conventional commoning, this framework makes it possible to evaluate whether, as we suspect, heterotopic commoning (which we expect to see where there is evidence of “denormalizing mutualization”) is more likely to be transformative, while non-heterotopic or conventional commoning (which we associate with “normalizing mutualization”) is less likely to stimulate transformation and may even support the status quo. This article offers a proof of concept for our framework. More extensive empirical work with this framework has the potential, we believe, to offer important insights into the possibilities for transformative commoning and the conditions that support it.

The rest of the article proceeds in four steps. First, we elaborate on our understanding of commoning and heterotopia and their relationship to social transformation, understood as systemic change in relationships, not only among people, but between society and the economy, ecology, and authority. Second, building on this conceptual work, we present our framework and strategy for evaluating relationships between commoning and heterotopia. Third, we draw on examples from contested urban green spaces in Belgium and Canada to illustrate how the framework can guide empirical research. Finally, we reflect on the potential contributions and limitations of our framework for advancing our understanding of commoning, heterotopia, and their relationships to social transformation.

### Commoning and heterotopia

The term “commoning” was rarely encountered outside of historical scholarship on medieval European commons until Linebaugh (2008) made the case for its contemporary relevance. Its emphasis on social practices, processes, and social ecological relationships distinguishes commoning from several related concepts, including “the commons,” which refers to a place, space, or thing that is shared (e.g. Hardin, 1968); common property, a collective form of private property (Ciriacy-Wantrup and Bishop, 1975); and common-pool resources, which are economic goods characterized by rivalry in consumption and difficulty of exclusion (Ostrom, 1990; Ostrom and Ostrom, 1977). Through commoning, people figure out how to “get along with each other” (Bollier and Helfrich, 2019: 75) and develop “affective socionature relations that can foster subjectivities of ‘being-in-common’ with others” (Singh, 2017: 754).

Scholars who embrace the concept of commoning agree that it gives rise to commons. There is, however, no consensus on whether commoning refers to practices or processes. Singh (2017: 755), for example, focuses on “practices of commoning”; Agrawal et al. (2023) define commoning as “shared, collaborative, situated practices through which groups of people with joint goals create the commons” (p. 544; cf*.* Nightingale, 2019: 16). Sevilla-Buitrago, on the other hand, defines commoning as “the process of shaping communal spaces, relations, and imaginations” (2022: 19), while Gibson-Graham et al. (2016: 195) suggest that it is “a relational process—or more often a struggle—of negotiating access, use, benefit, care, and responsibility.”

The distinction between practices and processes is subtle; in some cases, it may reflect nothing more than a stylistic choice. Velicu and García-López (2018), for example, define commoning as “the social practices engaged in re-claiming and sustaining the collective reproduction of commons” (p. 56), but then refer repeatedly to “commoning processes.” Yet, as we discuss further in the next section, the relationship between practices and processes is context-specific rather than fixed. Many different practices may contribute to the same process, while practices that support a process in some situations may undermine it in others (Tilly, 2001). Researchers can observe activities and practices (or their traces), or learn about them through ethnographic research; indeed, determining how specific practices influence specific processes requires interpretation grounded in specific contexts. Thus, we distinguish between practices and processes to define commoning as a set of processes that may or may not be compatible with diverse practices. More specifically, we understand commoning as a set of processes through which a set of people come to recognize that they share a place, space, thing, or project and to develop a sense of mutuality toward each other and whatever it is that they share (Poteete et al., 2021). Recognition here does not imply that all participants in commoning know one another as individuals. Commoning regularly involves strangers, such as people who share urban spaces (Arampatzi, 2022; Huron, 2015), participants in occupations and alternative economies (Alam and Houston, 2020; Varvarousis and Kallis, 2017), and contributors to various crowdsourcing projects and platforms (e.g. Wikimedia commons—see, e.g. Hammwöhner, 2013). Commoning does not require every participant to recognize every other participant, but it does require a recognition that there *are* others with whom one shares something. Moving from recognition to developing a sense of mutuality entails the development of relations of reciprocity either after noticing interdependencies among those who share something or deciding to create such interdependencies.

Heterotopia is more difficult to pin down. The term literally means “other” or “different” places. As introduced by Foucault (2004 [1967]) in absolutist terms, “otherness” suggests deviance and marginality or sacredness, while also being off-limits—yet Harvey (2009: 161) was not alone in critiquing this approach, lamenting how this essay had been widely taken up “as somehow definitive of ways to define liberatory spaces.” Lefebvre (1971), for instance, suggested that it is more useful to speak of “contrasting” spaces, which Hetherington (1997) specified as those with alternative orderings that are often ambivalent in their relationships to other sites. We accordingly invoke heterotopia in ways that are more polyvalent. If, as Foucault (2004 [1967]) first suggested, spaces deemed “heterotopic” are often associated with cultural otherness or marginality—that is, sites or situations where accepted social practices deviate from the norm, including places to which activities that (might) contest or reverse the everyday normalizations of dominant social practice get banished (such as carnival grounds, prisons, and mental asylums)—Lefebvre's (1971, 1974) more generous approach highlights their potential for transformation in some or indeed all other spaces, thus existing in generative tension with both dominant (“isotopic”) spatial orders and idealized (“utopian”) spaces of expressive desire (Harvey, 2009). A minimalist approach could acknowledge heterotopia as a space marked by limited to nonexistent formal programming or official presence.

The ambiguities that make the concept of heterotopia both compelling and frustrating feature regularly in descriptions of urban spaces as heterotopic. As ideal typical “other” places, heterotopias defy norms and are associated especially with marginal spaces (Dehaene and De Cauter, 2008; Doron, 2008). These include the outskirts of metropolitan areas (the rural–urban fringe), areas adjacent to transport infrastructure (e.g. expressways and railways), transition zones between areas with distinctive formal uses (e.g. industrial, commercial, and residential), “vacant” lots or buildings, deindustrialized zones, and socioeconomically marginal spaces. Many scholars in urban studies have thus cross-fertilized Foucault's conceptualization with Lefebvre's counter-definition, embracing notions such as looseness (which emphasizes flexibility undergirded by a lack of dominant formal programming—see Franck and Stevens, 2007), and healthy forms of porosity vis-à-vis imaginaries and social boundaries (Stavrides, 2007).

Working with a term that encompasses both highly regimented spaces dedicated to the repressive management of threats to hegemonic social norms and spaces that may seem anarchic (i.e. with no set purpose) does pose analytical challenges (Lees, 1997). It also addresses a compelling puzzle identified by Harvey (2009: 162): the political problem of finding ways “to realize their ephemeral potentialities in the face of powerful forces that work to reclaim them for the dominant praxis.” Our understanding of heterotopia emphasizes two dimensions that, as we discuss below, create opportunities for transformation: a lack of definition that allows for multiple coexisting imaginaries and ways of being, and either ambivalence about or change-focused attitudes (even antagonism) toward existing norms.

As noted above, where *the commons* can be used to describe things, spaces, or places, *commoning* refers to processes related to shared things, spaces, places, or projects. Likewise, we distinguish between *heterotopia* understood as ideal typical spaces characterized by looseness and a (transformative) lack of concern for norms, and *heterotopic processes*, which are generative of those conditions—namely loosening on the one hand and denormalization on the other. Loosening contrasts with “tightening”—that is, top-down programming that defines the identity of spaces and specifies their uses (Franck and Stevens, 2007). Loose spaces are ambiguous, vague, and liminal, sometimes lacking an agreed-upon identity or function. The open-endedness of what constitutes a loose space and what it is “for” allows the proliferation of multiple coexisting imaginaries. Denormalization may result from a simple lack of regard for norms or from intentional circumvention and undermining of dominant norms.

Our understanding of how heterotopic processes create opportunities for transformation parallels claims made by Varvarousis and Kallis (2017)—that is, commoning is transformational when it occurs in liminal conditions. Conceptually, heterotopia overlaps with or even subsumes liminality, understood as transitional or threshold spaces (e.g. Stavrides, 2007). Liminality, however, implies an unmooring or disruption that occurs to varying degrees in heterotopic spaces. We contend that in the absence of events strong enough to unmoor the identities of people and places (i.e. crises), processes of loosening and denormalization may nonetheless create opportunities for social transformation. Consequently, if they do not require unmooring, disruption, or crisis, heterotopic processes may occur in a greater variety of contexts than liminality (or at least with this understanding of it).

### A framework for analyzing commoning and heterotopia

The multidimensionality and socially constructed nature of commoning and heterotopia present challenges for analysis. To address these challenges, we propose an analytical framework that focuses on the association of commoning and heterotopia with two intersecting yet distinct processes: individualization–mutualization and normalization–denormalization. Although mutualization and denormalization are not the only processes associated with commoning and heterotopia, we contend that they are especially important for fostering social transformation.

Social transformation refers to “systemic change in institutions and social relationships, social norms and values, and relationships of power” (UC Santa Cruz Institute for Social Transformation, 2025). It can only be evaluated with reference to the status quo in a particular context. What counts as socially transformative in one time and place may support the status quo in another. Furthermore, history is neither linear nor inherently progressive, in part because social relationships, norms, and values are polyvalent. Some aspects of the status quo may be reinforced at the same time as others are transformed. In the analysis that follows, we assess social transformation with reference to a status quo characterized by individualism, commodification and financialization, hierarchical relations of authority, and a prioritization of economic growth over ecological wellbeing, all of which are typical of postindustrial cities in Western Europe and North America. We do not presume to know what social transformation might yield.

According to our working definition, commoning involves recognition of others who share something, followed by the development of a sense of mutual responsibility toward each other and whatever is shared. The transformative potential of commoning in contemporary capitalist societies, at least in postindustrial urban settings, does not flow from the process of recognizing things as being shared, but rather from the process of mutualization. As Linebaugh (2008: 103) observed, “[t]he allure of commoning arises from the mutualism of shared resources … Reciprocity, sense of self, willingness to argue, long memory, collective celebration, and mutual aid are traits of the commoner.” Commoning presents a clear alternative to individualism in mainstream contemporary society because it entails mutualization. Mutualization presents a direct challenge to hierarchical relationships, contributes to decommodification (Arampatzi, 2022; Caffentzis, 2010; Federici, 2019), and fosters changes in subjectivities, imaginaries, as well as affective relations that favor social embeddedness and, at least in some contexts, respect for the more-than-human (Dombroski et al., 2023; Singh, 2017; Varvarousis and Kallis, 2017).

If commoning involves mutualization by definition, it has a complex relationship with normalization–denormalization. As a sense of mutual responsibility emerges, so do informal norms. Norms associated with commoning sometimes clash with broader societal norms, but commoning is not inherently counter-cultural (Tummers and MacGregor, 2019; Velicu and García-López, 2018). Mutualization, for example, does not guarantee equity, inclusivity, or sustainability. Indeed, it can reinforce long-standing patterns of inequality (Kashwan et al., 2021; Nightingale, 2019) and is susceptible to incorporation into hegemonic processes (Anderson and Huron, 2023; Helten, 2015). The mutualization associated with commoning also sometimes effectively subsidizes capitalist relations, reinforcing the status quo by making it more palatable (Caffentzis, 2010). Thus, commoning can contribute to normalization *or* denormalization.

We identify heterotopia with looseness and denormalization. Both are potentially supportive of social transformation. Looseness enables a proliferation of imaginaries and ways of being, some of which—including commoning—are potentially transformative. Denormalization is necessary for social transformation, but it is not sufficient. Lees (1997) has, for example, shown how heterotopic spaces can both support and undermine dominant systems and hegemonic norms. Whether denormalization is transformative depends among other things on its substantive import, long-term effects, and potential to expand beyond the margins. Our focus on denormalization reflects our expectation that the extent of denormalization strongly influences both the occurrence of periods of liminality and responses to looseness.

Just as commoning relates to normalization–denormalization in diverse ways, heterotopia has no particular association with individualization–mutualization. For some, denormalization may suggest individualization: persons acting in ways that reflect their own needs or desires, including the desire to distinguish oneself, rather than social relationships. Yet subcultures, communities, organizations, and other sorts of collectivities also resist assimilation and homogenization, thus contributing to denormalization. Our analytical framework allows for both possibilities.

**Figure 1.**
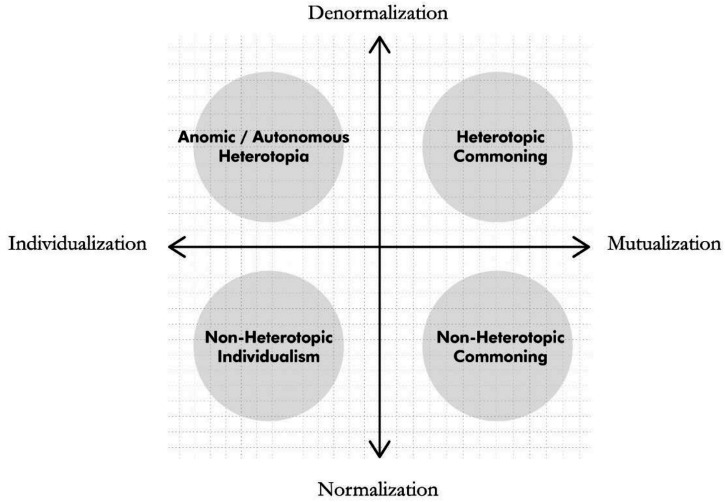
Matrix of individualization–mutualization and normalization–denormalization.

The two dimensions of our analytical framework, individualization–mutualization (commoning) and normalization–denormalization (heterotopia) form a matrix that defines four quadrants (see Figure 1):

- denormalizing mutualization, expected to be associated with heterotopic commoning;
- normalizing mutualization, expected to be associated with non-heterotopic or conventional commoning;
- normalizing individualization, expected to be associated with non-heterotopic or conventional individualism; and
- denormalizing individualization, expected to be associated with heterotopic individualism.

**Figure 2.**
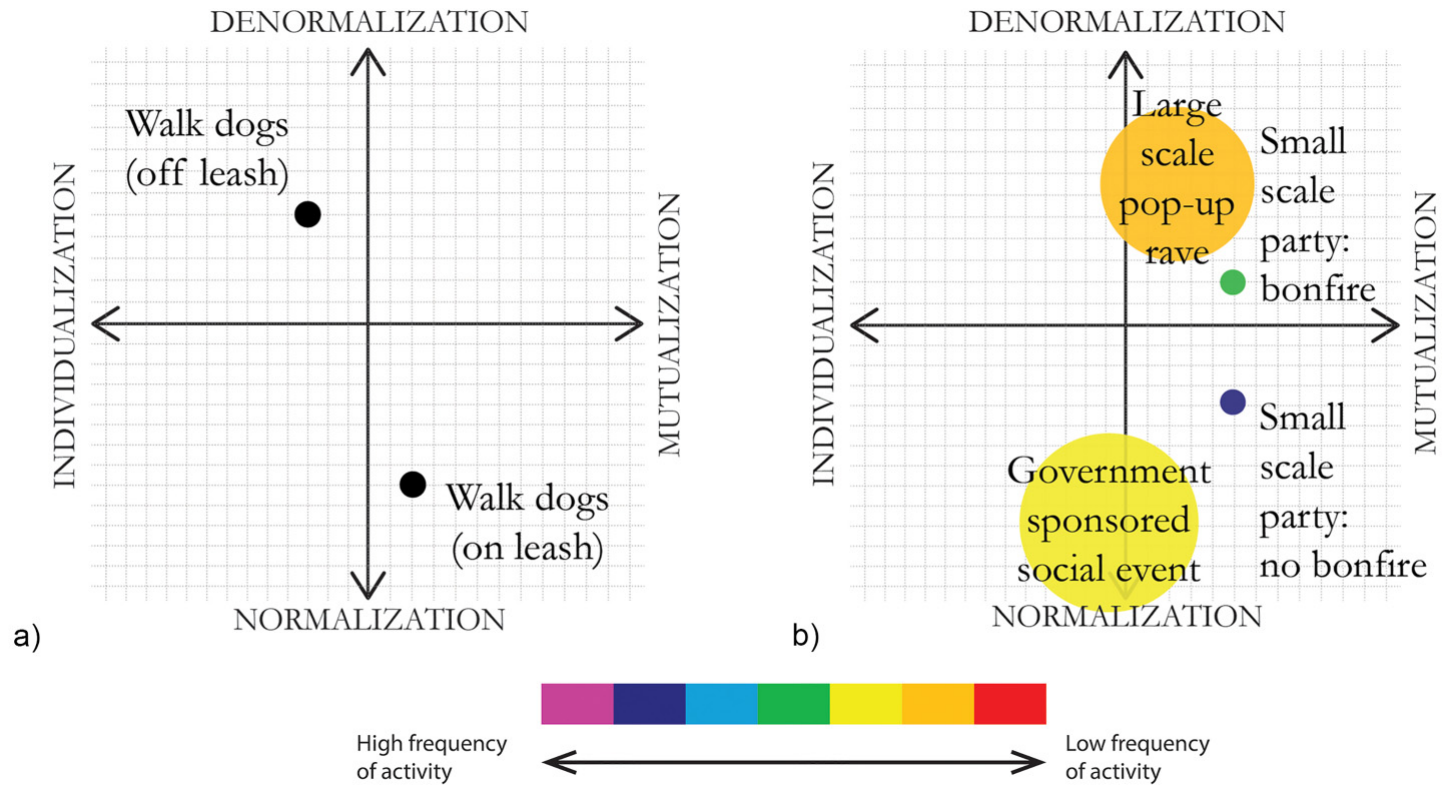
Applying the matrix: considering activities in context. (a) Walking a dog on- versus off-leash and (b) parties and social events with varied frequency and organizers. *The size of dots reflects the relative size of activities. Colors indicate their relative frequency, as indicated on the rainbow scale.*

It is important to note that the associations we propose may or may not be realized in specific cases because, as we have noted above, commoning involves more than mutualization and heterotopia involves more than denormalization. Activities taking place in a particular setting during a particular period can be located in this two-dimensional field, enabling analysis of their distribution across the four quadrants and whether and how that distribution changes over time. We do not want to suggest a reductionist approach. Indeed, the implications of a specific activity for normalization–denormalization and individualization–mutualization depend on the context in which it occurs. Consider the examples of dog walking and partying (see Figure 2). In general, walking a dog might be perceived as a normalizing activity with no bearing on mutualization. To the extent that walking a dog creates opportunities for developing relationships, however, it may contribute to community-building, which may in turn support mutualization. Walking a dog off-leash, on the other hand, challenges social and/or regulatory norms in many contexts and, if it ignores concerns expressed by others, may be perceived as a self-centered, individualistic practice. Likewise, social gatherings reinforce or undermine widely held norms and thus contribute to or threaten mutualization to varying degrees. When large-scale outdoor raves became more common during the COVD-19 pandemic, for example, they often challenged local as well as broader societal norms, even if they fostered a sense of community among participants. Scale is not the only issue. Large outdoor parties may be highly normalizing when they are sponsored or curated by local governments, transforming a “carnival” activity (spontaneous and popular, in effect) into a “festival” organized for tourism and economic development (Luka et al., 2015). Likewise, parties with bonfires (regardless of their size) challenge hegemonic norms in many contexts and create risks for nearby communities. We use this framework below to guide preliminary assessments of whether, as we suspect, the prospects for transformation are greater when commoning is heterotopic in three urban cases.

### Analyzing commoning, heterotopia, and social transformation

We illustrate our framework in three urban contexts: La Chartreuse in Liège (Belgium), the Champ des Possibles in Montréal (Canada), and Josaphat in Brussels (Belgium). La Chartreuse is a former military installation, while the Champ des Possibles and Josaphat are both former railyards. As they regenerated ecologically, all three sites attracted heterotopic activities and, eventually, plans for new urban projects. Commoning also emerged in all three sites as people mobilized to prevent the destruction of these open green spaces in the name of development, but it has varied in its relationship with heterotopia and its transformative effects to date. These cases are examples of a broader phenomenon of open spaces with heterotopic qualities that have recently become focal points for commoning movements. Other well-known examples include Berlin's former Tempelhof airport (Hilbrandt, 2017) and, in France, the Zone à défendre (ZAD) at Notre-Dame-des-Landes (Florez et al., 2022; Pruvost, 2017),^1^ the cigarette factory called La Belle de Mai in Marseille (Della Casa, 2013), and the Saint-Vincent-de-Paul hospital in Paris, better known as Les Grands Voisins (Encore Heureux, 2018).

Guided by our framework, we sketch changes in the relative prominence of and interactions between commoning and heterotopia over time for each case and offer preliminary assessments of the implications for social transformation. As we show below, activities associated with commoning ranged from quite conventional to highly denormalizing and, while all three cases experienced periods of both heterotopic and non-heterotopic commoning, the degree of denormalization appears to vary across sites. We first present La Chartreuse—where commoning seems to have been the most denormalizing—and end the section with Josaphat, where heterotopic commoning apparently remained nascent. These sketches provide preliminary support for our expectation that heterotopic commoning has greater transformative potential than non-heterotopic commoning.

Our characterization of various activities along the dimensions of individualization–mutualization and normalization–denormalization, as well as our characterization of dynamics during particular periods, both reflect our qualitative understandings of the social significance of various activities in each context. These evaluations are inevitably subjective, and our own perspectives and values differ. Coming to common assessments involved considerable debate, during which we weighed support for alternative interpretations based on our research and personal experiences.^2^ More specifically, our analysis draws on documentary research, direct (nonparticipant) and participant observation, and informal conversations with passersby. For our Montréal case, we also conducted semistructured interviews and focus-group discussions.

#### La Chartreuse: A burst of highly heterotopic commoning?

The site of a decommissioned military installation, La Chartreuse is a 40 ha forested hill now subdivided into lots, some of which are owned by the municipality of Liège and some by two real-estate development companies (Snyers, 2019). Around 1990, part of the site was acquired by the city government and protected as the Parc des Oblats (Snyers, 2018). An 11 ha portion, however, was designated as a ZACC (Zone d’aménagement communal concerté),^3^ and earmarked for new construction. With the exception of some nineteenth-century military barracks, the municipality demolished all structures on this portion in 2008 (Michaux, 2018). For years, the park was intentionally managed in a loose “rewilding” manner to promote biodiversity under a special provision (Ruelle and Lefebvre, 2023).^4^ Over time, La Chartreuse also attracted marginalized populations and activities, soon gaining a reputation as a refuge for people experiencing homelessness, a hub for street art and urbex photography, and a place for sunbathing, nature appreciation, dog walking, and music. These activities often involved individuals and small groups who appreciated the loosening of dominant social norms in this space.

Change loomed, however. In 2017, one of the private firms requested authorization to construct a 74-unit residential development on the ZACC, triggering grassroots opposition (Snyers, 2019).^5^ Local residents and others opposed to these plans formed a nonprofit organization, Un Air de Chartreuse, to advocate for protection of both the ZACC and the park as a contiguous common space that was green, biodiverse, and peaceful.^6^ Despite some adjustments to the development plans in response to demonstrations and petitions, opposition to any new construction persisted until 2020, when the COVID-19 pandemic halted both the commoning process and urbanization plans.

The pandemic was an important moment that allowed the multiplication of heterotopic activities. Free weekly parties were held illegally on the site in 2020, attracting punk, queer, and anarchy-oriented communities, which fostered widespread knowledge of the site while also increasing its daytime use. When planning for construction resumed in 2022, people who had discovered the site through heterotopic activities joined forces with conservationists to form a collective, Chartreuse occupée (occupied Chartreuse), to defend the site. The group occupied the ZACC for 8 months, adopting tactics inspired by the French ZAD movement. These included assemblies, artistic interventions, readings, parties, the erection of barricades, and anticapitalist and ecological messaging, which took place not only on-site, but also through conventional news and social media. These activities attracted disparate publics, from participants in pandemic-era heterotopic activities and ZADists from far and wide, to residents concerned about local futures.

In October 2022, Chartreuse occupée, the private developer, and the city mayor agreed to terminate construction and instead integrate the ZACC into the Parc des Oblats as a public open space. This agreement was two-fold: (1) the private developer would not pursue development of their property in exchange for a (not-yet-public) agreement with the municipality for development in another part of the city and (2) Chartreuse occupée would cease its occupation quickly and return the site to its former state by, for example, dismantling shelters and barricades, and removing accumulated material (Demeyer, 2022). With reassurance that the site would remain a relatively loosely managed green space, the mobilization quickly dissipated. Once the intense yet successful period of occupation ended, dynamics quickly shifted back to what we see as conventional individualism ruled by a regime of individual responsibilities and concerns. Un Air de Chartreuse resumed its role as the primary interlocutor with the city administration. Other than Un Air de Chartreuse's annual clean-up day and its recent mobilization to protest destruction of vegetation following a film shoot on the site (Un Air de Chartreuse, 2025), there have been few commoning activities since the successful mobilization in 2022.

This case illustrates how disparate actors coming together in mutualizing and denormalizing activities can give rise to transformational commoning and reshape a space's future. Prior to the pandemic, Un Air de Chartreuse had pursued more-or-less conventional commoning, involving mutualization, but also normalizing activities, such as enjoying nature and quietness, and conventional forms of lobbying, such as petitions, with limited results. During the pandemic, free illegal parties and other heterotopic activities challenged dominant societal norms but generated few collective claims. More substantial change occurred after these diverse actors joined forces and occupied the space for an extended period. Only then did public officials adopt policies to protect the site. One could argue that developments since the occupation demonstrate the unstable nature of both denormalizing mutualization and transformative commoning. They also demonstrate the potentially placating nature of institutional agreements, with the return to a status quo deactivating both collective claim-making (mutualization) and denormalizing forms of mobilization. On the other hand, the occupation engaged strangers from highly diverse backgrounds in denormalizing activities associated with heterotopic and transformative commoning over a period of several months. It remains to be seen whether these experiences transformed participant subjectivities, thus increasing the likelihood of transformative commoning in other contexts in the future.

#### Champ des Possibles: From heterotopic to conventional commoning

Our second site is an abandoned railyard in the central Montréal neighborhood known as Mile End. The Canadian Pacific (CP) Railway built a trunk line adjacent to the site in the 1870s and the one-hectare railyard became an anchor for local industries until its official closure in 1976 (Desjardins, 2019a, 2019b). The railyard's closure created opportunities for revegetation and the emergence of denormalizing—or what we characterize as heterotopic—individualism. As with La Chartreuse, the former railyard offered a refuge for people experiencing homelessness and various other marginalized individuals and subcultures. It became an illegal dumping ground but also a space for art installations, social gatherings, raves, and bonfires. Pedestrians regularly passed through the site as a convenient shortcut, often illegally crossing the railway, on which a handful of freight trains still operate each day.

A period of denormalizing mutualization seems to have started after the city purchased the site from CP in 2005. As a part of their plans to “revitalize” the broader sector, local authorities explored the possibility of extending an abutting cross-street through the former railyard, which in turn would become a paved yard for storing public-works vehicles. In 2007, artist Emily Rose Michaud responded by creating the Roerich Garden, a large-scale landscape art installation crafted on the site by fellow artists and local residents using natural materials such as mulch and rock. The resultant 30 m^2^ Roerich symbol identified the site as a threatened cultural resource in need of protection.^7^ Subsequently maintained by the Sprout Out Loud!/Le Pouvoir au Pousses!collective, it became a focal point for workshops, picnics, bonfires, seed exchanges, and guerrilla gardening, all explicitly intended to (re)claim the space for ongoing acts of commoning (McSwiney and Michaud, 2014: 272).

Official announcement of the local government's plans in 2008 gave rise to Mile End en chantier (Mile End under construction), a broader, multifaceted mobilization spearheaded by the Mile End Citizens’ Committee (MECC). Through MECC's public assemblies and workshops, those involved in the Roerich Garden connected with others who shared their desire to maintain the former railyard as a green, but also relatively loose, communal space. A coalition of artists, ecologists, urbanists, anarchists, and residents coalesced around these goals and named the space the Champ des Possibles (a French idiom for the realm of the possible). They mobilized broad support for prioritizing its recreational, environmental, and artistic potential through means similar to those initiated by Michaud (e.g. participatory workshops, picnics and parties, artistic interventions, and ecological tours).

A period of institutionalization followed this short burst of what we consider to be heterotopic commoning. Prior to the 2009 municipal elections, MECC organized a debate at which participants in the Mile End en chantier process—including those advocating protection of the Champ—pressed candidates to respect priorities defined through a yearlong process of decentralized, open discussions. A relatively new progressive party promised to do just that and won every seat on the local council. In 2010, the new council canceled the contested development project and commissioned a new plan featuring the Champ des Possibles, not as a park, but as a protected public green space (Atelier BRAQ, 2010). The council also initiated discussions with city officials to win support for co-managing the space in a relatively loose manner. Both ideas were unheard of in Montréal; gaining approval for these novel approaches took some time. Because the local council could not enter an agreement with a loose coalition, participants in the mobilization to protect the site established a nonprofit organization called Les Amis du Champ des Possibles in 2010. The site was designated as a protected natural space in 2013 and the council signed a three-year partnership agreement with Les Amis for its co-management in 2014 (Plateau-Mont-Royal and Les Amis du Champ des Possibles, 2014). This agreement was subsequently renewed in 2017 and 2022 and, as of 2025, remains in force. As part of the partnership agreements, the local government provides Les Amis with a modest operating budget. These developments ushered in a certain hegemony and a dynamic of normalization. Formally recognized as co-managers of the site, with a government-financed coordinator, Les Amis increasingly took a leading role in defining priorities for the Champ and overseeing its day-to-day management. The organization asserted greater control over the site by, among other things, removing or destroying earlier installations (e.g. flower boxes and a simple stage for performances), cordoning off zones within the site for restoration, reducing the number and width of footpaths, and discouraging informal bonfires.

Between 2007 and 2010, activities in the Champ des Possibles involved denormalizing mutualization, corresponding to what we call heterotopic commoning. These processes not only blocked normal planning priorities; they also led to transformative changes to Montréal's approach to urban green space, including the city's first co-management agreement, its protection of a postindustrial open space as a natural area, and the adoption of a minimal-intervention approach to its management. The Champ has inspired residents across the city of Montréal to mobilize to defend other postindustrial or otherwise “vacant” spaces. Neither a park nor a well-defined public space in any conventional sense of the term, the Champ stands in stark contrast to other carefully controlled public spaces in Canadian cities. With institutionalization and co-management, however, this apparently heterotopic commoning gave way to normalizing mutualization, which we see as conventional commoning, with a prioritization of biodiversity protection over social “wildness.” Heterotopic activities continue to take place in the Champ but no longer support large-scale commoning. Meanwhile demobilization, especially during the pandemic, has made it difficult to sustain any form of commoning. The situation as of 2025 might best be described as an uneasy cohabitation of what we consider to be heterotopic individualism and conventional commoning.

#### Josaphat: Mostly conventional commoning?

Participation in heterotopic processes extends beyond those attending free illegal parties, anarchists, or socially marginalized publics. In the case of the abandoned Josaphat railyard in Brussels, the heterotopic activities of naturalists eventually gave rise to what appears to be non-heterotopic commoning. The 24 ha railyard had closed in 1994 and was left to rewild by public authorities in 2012 in anticipation of its transformation into a large “eco-neighborhood” (Sauvons la Friche Josaphat, 2023). As it regenerated, ornithologists and other naturalists increasingly (illegally) frequented this vast open area to watch birds and wildlife and to conduct inventories of species. These (slightly) denormalizing activities were uncoordinated and devoid of any collective agenda; the naturalists were merely enjoying ephemeral conditions created by delays in the process of land development, with no intention of intervening in that process. We consider this to be a period of mildly heterotopic individualism.

The evidence of biodiversity compiled by naturalists reinforced the efforts of several activist organizations who established a citizen collective called Commons Josaphat in 2012 (Leclercq, 2023). While the activities of Commons Josaphat promoted mutualization and called into question several dominant norms, the collective relied primarily on conventional political strategies. At first, this initiative lobbied for modifications of the “development” project to favor community-oriented sustainability (including collective spaces and services, shared and intergenerational housing, high-performance construction, preservation of some natural areas). The collective organized a series of on- and off-site workshops focused on shared and tiny housing, urban agriculture, open-source knowledge, and community land trusts. These activities culminated in the publication of an explicit manifesto for the site, including arguments for more thoughtful planning to conserve and enhance existing biodiversity (Commons Josaphat, 2015). Suggestions included the daylighting of buried watercourses, enhancing existing vegetation, and a redefinition of the areas to be densified with the goal of increasing biodiversity protection. While the workshops and manifesto promoted mutualization and raised questions about dominant norms, the collective did not challenge existing processes of planning or decision making. In our assessment, normalizing processes outweighed denormalizing processes, limiting the transformative potential of commoning during this period.

While local authorities expressed support for the collective's initiatives, representatives of Commons Josaphat found that their work had little transformative impact on the building projects, especially after 2019, when the Brussels Region adopted a strategic plan for the area that was based largely on previous schemes dating from 2013. Angered that their proposals had not influenced the new plan, the collective ended its involvement with the municipality in protest, refusing to “keep playing this show written by the government,” as stated by Tom Lootens, leader/spokesperson for one of the civil-society groups (Leclercq, 2023: 145). In 2019, members of Commons Josaphat and naturalists formed a new collective, Sauvons la Friche Josaphat, which demanded protection of the open space and its growing biodiversity and a scaling-back of the building project. They also continued to use conventional political strategies to advance this agenda. The city adapted its strategic plan for the area in response to renewed mobilization in 2021. It reduced the projected number of housing units from 1600 to 1200 and took a stronger stance on green spaces and biodiversity by concentrating the building footprints more strategically, tripling the amount of reserved open space. Nonetheless, the 2021 plan earmarks only 1.4 ha of the 12 ha open area for protection. Commoners remained dissatisfied with these changes and continued to demand stronger conservation of the area. Several organizations worked together to compile three alternative scenarios in their “Plan B” for the site's future (BRAL and Natagora, 2021). Sauvons la Friche Josaphat then launched a petition opposing the project, which garnered 20,000 signatures by the end of 2021. Pressure on the developers increased the following year when, based on observations compiled by naturalists, the public organization Bruxelles Environnement designated Friche Josaphat as one of seven nonforested sites in the Brussels Region with “very high ecological value.” The ongoing debate created divisions within the governing coalition, especially between the Socialist Party, which promoted the 2021 plan, and the Ecologist Party, which supported its revision. The issue even threatened the coalition's survival, a year before regional elections shifted the majority away from both parties and toward the right-wing Liberal Reformist Party.

In this case, Commons Josaphat, Sauvons la Friche Josaphat, and other organizations mainly engaged in what can be seen as conventional commoning, with incursions into the realm of denormalization. Commoning built on the apolitical, uncoordinated, and somewhat heterotopic activities of naturalists, which produced evidence of the value and richness of the site's urban biodiversity (the strongest argument in favor of its protection). This argument was first embraced by urban activists within a larger movement to develop the site in a collective and ecologically responsible manner, and then by the public administrators and designers responsible for the project, who (partially) adapted the plan. In 2023, despite the risk of political instability, part of the governing coalition pushed for revision of the plan to strengthen biodiversity protection. In contrast with the other two cases, however, there has yet to be a real convergence of denormalization and mutualization, of heterotopia and commoning. In other words, heterotopic individualization generated a motivation for commoning, but it has not fed into it. The scenarios proposed in the Josaphat “Plan B” (BRAL and Natagora, 2021), while provoking intense debates about the current plan, entail rather normalized functions and social practices (e.g. biodiversity conservation, leisure, and new housing). At least to date, commoning seems to have limited transformative potential.

### Reflections

This article introduces a framework to guide empirical analysis of the relationships among commoning, heterotopia, and social transformation. We have presented three cases to show how our framework can support empirical research on the relationship between commoning and heterotopia in particular spaces and periods of time, specifically for analyzing relationships among commoning, heterotopia, and social transformation. The cases suggest that, as argued by others (Anderson and Huron, 2023; Tummers and MacGregor, 2019; Velicu and García-López, 2018), while commoning has transformative potential, it is not inherently or inevitably transformative. A first step toward improving our understanding of the relationship between commoning and transformation is to identify other processes or conditions that might interact with or mediate the effects of commoning, such as heterotopia. By way of conclusion, we first reflect on our framework: how it influenced our analysis as well as its contributions and limitations. We then summarize our empirical findings and highlight unsettled theoretical and empirical questions about the relationships among commoning, heterotopia, and social transformation.

#### Varieties of commoning

Perhaps akin to the actions of heterotopic commoners, a valuable framework is one that changes what we see. Our framework does exactly this. It brings into focus different forms of commoning and heterotopia, giving rise to new questions about when and how these phenomena vary and change and how, in combination, they may transform a piece of land, a local community of people, and/or the political and economic situations into which they are sometimes forced. While various processes can be associated with commoning and heterotopia, we contend that mutualization and denormalization are especially likely to foster transformation. The two-dimensional conceptual space defined by individualization–mutualization and normalization–denormalization distinguishes two varieties of commoning, as well as two scenarios in which commoning is absent. The framework provides an analytical language for describing all four possibilities: non-heterotopic or conventional commoning, heterotopic commoning, heterotopic individualism, and conventional individualism. It thus draws attention to and enables structured comparisons of what can be characterized as heterotopic and non-heterotopic commoning, as well as comparisons of dynamics when commoning, regardless of variety, is present or absent.

The framework provided coherent conceptual tools and an analytical language to interpret our case material. Behind the scenes, we plotted activities in particular places and periods on two-dimensional charts akin to those in Figure 2. We used visualization tools such as color-coded circles of varying size to depict differences in the scale and frequency of various activities to highlight the distribution of activities across the four quadrants and to track changes over time. Our characterization of spaces and periods reflects the quadrant in which most activities cluster. In other situations, activities might cluster in half of the matrix rather than within a single quadrant, or there may not be any dominant dynamic. We expect this framework to facilitate comparative research on the relationship between commoning and heterotopia, on factors that influence that relationship, and the implications for social transformation and other outcomes of interest.

As with any other framework, ours has limitations, four of which we briefly discuss here. First, it considers only two of the many processes that contribute to commoning and heterotopia. We acknowledge that this strategy limits the framework's applicability but note that focusing on a limited number of processes and dimensions can be a strength if they are especially important for the outcome of interest. Second, application of the framework relies on subjective assessments of the context-specific significance of activities. Participatory and ethnographic research can help researchers understand the local context. Yet even similarly well-informed scholars may assess the same activities differently, reflecting differences in positionality as well as in perspective. We see the framework as encouraging reflexivity, which can reduce if not eliminate subjective biases by prompting researchers to reflect on how particular activities interact with social processes, and how contextual factors influence those interactions. This includes possibilities for thinking more broadly about heterotopia as dynamic (Lefebvre, 1971, 1974), even if this includes some transformative normalization. In our experience, comparing and debating the assessments of colleagues with distinctive perspectives further guards against bias. A third limitation is that we designed the framework to evaluate the presence and variety of commoning associated with a space, not to identify all relevant actors with an interest in that space or to evaluate their influence. Commoning associated with a space, however, need not take place (solely) in that space. While occupations, workshops, recreational activities, dumping, and care work, among many other activities, have all taken place within one or more of our study sites, many other activities occurred elsewhere (meetings, outreach activities, and encounters with politicians and municipal staff). Our analyses take into consideration both on-site and off-site activities. More seriously, possibilities for social transformation depend not only on the presence and form of commoning, but also on the political economic context in which it occurs as well as its influence relative to forces that reinforce the status quo (Anderson and Huron, 2023; Arampatzi, 2022; Huron, 2015). Rather than adapt our framework to analyze the activities of economic, state, and other external actors, we prefer to embed analyses of bottom-up dynamics guided by our framework within a broader social, ecological, and political-economic analysis. Our framework does not do everything—nor should it!

The importance of context, however, points to a fourth limitation: the lack of diversity in our cases, all of which concern mobilization to block the (re)development of postindustrial green spaces in two wealthy countries. Our case analyses illustrate how changes in context—such as gentrification and electoral competition, changes of government or political coalitions, and shocks such as the COVID-19 pandemic—influence individual and social practices, how they are perceived and thus how they shape social processes, including commoning, heterotopia, and social transformation. Given the structural similarities among our three cases, however, we cannot assess the applicability of our framework in many other settings in the Global North, such as urban neighborhoods identified with specific cultural or socioeconomic communities or rural communities, much less in the Global South. While we believe that our framework has some degree of portability, some degree of cultural specificity (for example) in the processes associated with transformative commoning would not be surprising. Even if this specific framework proves to have limited relevance beyond postindustrial urban settings, we hope that it inspires more reflection on the processes that support commoning and how interactions with other processes and conditions mediate their transformative potential.

#### Commoning, heterotopia, and (social) transformation

We found preliminary support for our expectation that heterotopic commoning has more transformative potential than non-heterotopic commoning. In both the Champ des Possibles and La Chartreuse, short-lived bursts of heterotopic commoning opened windows for transformative change; as commoning became more conventional, commoners increasingly exerted influence by working within the existing systems of control. In the case of Josaphat, commoning with (relatively) weak heterotopic dynamics has not yet achieved significant let alone transformative change. It remains to be seen whether ongoing heterotopic commoning, even if limited, facilitates more expansive and transformative commoning in the future. Beyond demonstrating the plausibility of our argument, our analysis also raises new questions. We highlight four areas that warrant future research: (1) the instability of what we call heterotopic commoning; (2) the role of crises; (3) the role of intentionality in social transformation, and (4) the very ways in which social transformation gets conceptualized.

Our case material suggests that heterotopic commoning tends to be unstable, making it susceptible to both demobilization and normalization. Commoning supported the emergence of organizations that became regular interlocuters with local authorities, such as Un Air de Chartreuse in Liège, or even co-managers, like Les Amis du Champ des Possibles in Montréal. As these organizations developed closer relations with government officials, however, they seemed to either neglect commoning or lose their capacity to stimulate it. Accommodation by officials seemingly contributes to this process of normalization; “kindness” on the part of officials may “kill” the transformative potential of commoning—and even commoning itself (cf. Helten, 2015). The risks of cooptation and institutionalization to sustained mobilization are well-recognized (e.g. Arampatzi, 2022; Noterman, 2022; Varvarousis and Kallis, 2017: 151–153). We suspect that the normalization of commoning threatens the coexistence of multiple uncoordinated activities and imaginaries, which in turn undermines dynamism in general and may limit the potential for social transformation in particular.^8^ It is not clear, however, whether heterotopic commoning is inherently unstable or what the implications might be for social transformation. With reference to Lefebvre's (1971, 1974) comments on heterotopia often being in generative tension with both dominant (“isotopic”) spatial orders and idealized (“utopic”) spaces of desire, are there conditions under which a period of heterotopic commoning might achieve enduring transformative changes or set in motion transformative dynamics that could survive it?

Several recent studies suggest that crises create conditions favorable for commoning and consider whether they might lay the groundwork for transformative change (e.g. Anderson and Huron, 2023; Arampatzi, 2022; Dombroski et al., 2023; Varvarousis and Kallis, 2017). Our argument has some parallels with that of Varvarousis and Kallis (2017), who argue that commoning is most likely to be transformative during periods of liminality arising from catastrophic moments such as the 2008 financial crisis in Greece. Also associated with heterotopia, liminality is generally a form of denormalization that accompanies movement from one stable state to another. Crisis does not feature in our framework, however, and our findings are not consistent with the expectations of Varvaroulis and Kallis. Our study sites—two former railyards and a decommissioned military installation—are liminal in terms of having lost these older official functions. In each case, however, decades passed between this loss and the rise of commoning. To the extent transformative commoning occurred, it responded to threats that loose, denormalized spaces would be (re)incorporated into hegemonic processes of land “development.” In other words, commoning did not arise from crises resulting in liminality, but rather from crises threatening to destroy liminality. We do not doubt that crises sometimes prompt commoning and support social transformation, but crisis is neither necessary nor sufficient for transformative commoning (cf. Anderson and Huron, 2023).

Recognizing that the social import of a practice is always context-specific and socially constructed, our framework assumes that social transformation depends on the processes that practices reinforce or set in motion in a particular context rather than on the practices themselves. In the case of Josaphat, for example, birdwatching contributed to denormalization and mutualization, albeit unintentionally and modestly, while the efforts of Commons Josaphat and Sauvons la Friche Josaphat to denormalize and transform planning processes in Brussels have had limited effect thus far. Yet the cases of La Chartreuse and the Champ des Possibles demonstrate that practices adopted with the goal of fostering mutualization and denormalization sometimes succeed. Practices associated with Chartreuse occupée, the Roerich Garden, and Mile End en chantier intentionally stimulated mutualization and denormalization, and these processes in turn contributed to at least some degree of social transformation. These examples, in which one or a few individuals played a critical role in initiating commoning among strangers, support Noterman’s (2016) argument that, while “differentiated commoning”—commoning that is characterized by uneven participation—can undermine commoning, it can also play a supportive role in the survival and even the expansion of commoning (p. 449; see also Dombroski et al., 2023; Gillespie et al., 2018). In affirming not only the inadequacy of intent alone and the importance of unintended consequences, but also the possibility of intentionally inciting mutualization, denormalization, and social transformation, our cases raise questions about when and why efforts to initiate socially transformative processes enjoy significant success.

Finally, what counts as transformative change? Those who vaunt commoning's transformative potential typically contrast it with relationships and norms associated with contemporary capitalism. The normative contrasts are apparent, but the mechanisms of social transformation are less obvious. Leclercq (2023) argues that changes in narratives and imaginaries lay the groundwork for transformative change, while Dombroski et al. (2023), Gillespie et al. (2018), Singh (2017), Varvarousis and Kallis (2017), and Velicu and García-López (2018), among others, suggest that transformative change requires attitudinal and behavioral changes, which in turn foster fundamental changes in subjectivities. Ruling out a sudden and complete transformation as implausible means accepting that social transformation will be piecemeal and slow. Forms of commoning that are both highly visible and radically denormalizing, such as the sustained occupation of La Chartreuse, *seem* more likely than co-management to instigate changes in narratives, imaginaries, and subjectivities supportive of systemic social changes. Yet, in all the cases we investigated, transformation also came from more subtle, less-visible, and definitely less “radical” practices of negotiation with local authorities and profit-motivated land developers. In Montréal and Liège, those negotiations—enabled by highly visible denormalizing actions—yielded agreements for important changes, with their own caveats of institutionalization and what might be an entropic return to normalization. In noting that what we call heterotopic commoning clearly interacted with formal political processes to produce formal legal changes supportive of commoning in Athens and Madrid, Arampatzi (2022) suggests that this sort of institutionalization can support (and may indeed be necessary, albeit insufficient, to achieve) the scaling-up of transformative changes beyond the localities where they emerge. Perhaps social transformation requires the normalization of what was once denormalizing.

We close, therefore, with unanswered questions about the relationships linking different forms of commoning and heterotopia. Is heterotopic commoning more likely to emerge from conventional commoning or from heterotopic individualism? And, if heterotopic commoning is inherently unstable and cannot be sustained, what influences whether it gives way to heterotopic individualism, conventional commoning, or even conventional individualism? In turn, this approach also raises questions about the way these relationships affect social transformations. When are changes in narratives and imaginaries, attitudes, behaviors, and relationships significant enough to count as transformative? How might we evaluate changes in subjectivities? And how should we compare different types of social change? More research is required to better understand these relationships. We are confident that the framework introduced in this article not only provides the tools to recognize the presence of (changing) relationships between commoning and heterotopia but can also be used to evaluate the degree to which shifts in these relationships are associated with other conditions and processes, including changes in subjectivities and social transformation.

## Notes

1. Zone à défendre (Zone to be defended) designates a piece of land usually occupied by members of civil society seeking to block development plans. The acronym became famous after the successful battle against the construction of the Notre-Dame-des-Landes airport (France) from 2009 to 2018. ZADists refer to activists in this and similar occupations.

2. The first author, for example, has been a member of Les Amis du Champ des Possibles since 2018.

3. A Zone d’aménagement communal concerté (joint municipal development zone) is a Wallonian planning procedure that allows an area to be reserved for an as-yet unspecified function.

4. The site was subject to a Plan de gestion différenciée (differentiated management plan), a type of management plan used in Francophone countries to promote sustainable management practices in green spaces, including rewilding (see e.g. Du Toit and Pettorelli, 2019).

5. This was the first part of a wider plan for densification that involved the construction of a total of 400 housing units by 2038.

6. Unclear communication led some to believe that construction would take place in the park as well as in the ZACC.

7. The Roerich symbol, comprising three circles within a larger circle, was widely used in Europe during the World War II to identify hospitals and cultural heritage sites from above and thus protect them from aerial bombing. It is now associated with international law for the protection of cultural assets.

8. Tsing's (2015) conceptualization and discussion of polyphony offers an interesting way of thinking about the coexistence of multiple ways of being.
